# Maternal Embryonic Leucine Zipper Kinase (MELK): A Novel Regulator in Cell Cycle Control, Embryonic Development, and Cancer

**DOI:** 10.3390/ijms141121551

**Published:** 2013-10-31

**Authors:** Pengfei Jiang, Deli Zhang

**Affiliations:** 1 Research Laboratory of Virology, Immunology & Bioinformatics, Division of Veterinary Microbiology & Virology, Department of Preventive Veterinary Medicine, College of Veterinary Medicine, Northwest A&F University, Yangling 712100, Shaanxi, China; E-Mail: peng-feijiang@hotmail.com; 2 MOA Key Laboratory of Animal Biotechnology of National Ministry of Agriculture, Institute of Veterinary Immunology, Northwest A&F University, Yangling 712100, Shaanxi, China; 3 Investigation Group of Molecular Virology, Immunology, Oncology & Systems Biology, Center for Bioinformatics, Northwest A&F University, Yangling 712100, Shaanxi, China

**Keywords:** maternal embryonic leucine zipper kinase, protein interaction, cell cycle, embryonic development, tumorigenic process, therapeutic target

## Abstract

Maternal embryonic leucine zipper kinase (MELK) functions as a modulator of intracellular signaling and affects various cellular and biological processes, including cell cycle, cell proliferation, apoptosis, spliceosome assembly, gene expression, embryonic development, hematopoiesis, and oncogenesis. In these cellular processes, MELK functions by binding to numerous proteins. In general, the effects of multiple protein interactions with MELK are oncogenic in nature, and the overexpression of MELK in kinds of cancer provides some evidence that it may be involved in tumorigenic process. In this review, our current knowledge of MELK function and recent discoveries in MELK signaling pathway were discussed. The regulation of MELK in cancers and its potential as a therapeutic target were also described.

## 1. Introduction

According to studies on oncogenic signal transduction pathways, targeted therapies have made great progress in cancer treatment, whereas standard chemotherapy alone has failed to improve treatment outcome. In this paper, maternal embryonic leucine zipper kinase (MELK), a potential therapeutic target that is possibly involved in a tumorigenic process, was described. MELK, also known as murine protein serine/threonine kinase 38 (MPK38) [1] and Eg3 protein (pEg3) [2], was originally identified as a signal transduction factor by Gil *et al.* and Heyer *et al.* almost simultaneously [1,3]. MELK is also a cell cycle-dependent protein kinase that belongs to the KIN1/PAR-1/MARK family [2,4,5]. Unlike most members of this family only functioning in cell survival under metabolically challenging conditions [6–9], MELK participates in diverse processes, including cell cycle [4], cell proliferation [10], apoptosis [11,12], RNA processing [13], and embryonic development [3,14–16]. Furthermore, MELK is involved in multiple protein interactions that affect many stages of tumorigenesis [17]. Thus, this gene is potentially an effective therapeutic target.

## 2. *MELK* as a Regulator of Cell Fate

*MELK* is initially cloned in oocytes and detected both in normal tissues and in cancer cells. Evidence has shown that *MELK* is expressed by multipotent neural progenitors derived from different ages and necessary for the proliferation of such progenitors *in vitro*, thereby influencing their ability to form neurospheres, which indicate self-regeneration [10]. Previous studies have strongly indicated that *MELK*, unlike other *KIN1*/*PAR-1*/*MARK* family members, regulates cell cycle rather than cell survival. *MELK* is also required in the transition of proliferative glial fibrillary acidic protein (GFAP)-positive progenitor cells to highly proliferative GFAP-negative cells *in vitro*. These data have validated the important function of MELK in neural progenitor biology [10]. *MELK* is also expressed in proliferating myoblasts [18] and mammary progenitors [19], suggesting that *MELK* is commonly expressed in tissue-specific progenitor cells. A previous study using mice in various developmental stages as a model shows that the expression levels of *MELK* gene in retinal progenitor cells is positively correlated with their proliferative activities in mice; *MELK* also participates in the differentiation of glial cells [20], suggesting that *MELK* has multiple functions in retinal development.

In *Caenorhabditis elegans*, *PAR-1-like gene* (*PIG-1*) is identified as a worm ortholog of *MELK*. It is reported that *PIG-1* and *cell death gene-3* (*CED-3*) double mutants result in nearly 100% of lineages producing extra HSN and PHB neurons, leading to dramatically stronger programmed cell death than either single mutant, which suggests that *PIG-1* acts at least partly in parallel to the canonical cell death pathway [21]. *PIG-1* also regulates cell size asymmetry and asymmetric neuroblast divisions by controlling neuroblast polarity [21].

MELK physically interacts with apoptosis signal-regulating kinase 1 (ASK1), and the activity of ASK1 is either positively or negatively regulated by its interacting molecules, including tumor necrosis receptor-associated factor [22], Daxx [23], JNK/stress-activated protein kinase-associated protein 1/JNK-interacting protein 3 [24], thioredoxin [25,26], glutaredoxin [27], heat shock protein 72 [28], Raf-1 [25], Akt/PKB [29], PP5 [30], and 14-3-3 protein [31]. MELK, as an upstream kinase of ASK1, phosphorylates threonine 838 in an activation loop of human ASK1, thereby stimulating ASK1 kinase activity. Interestingly, this interaction enhances JNK-mediated transactivation and H_2_O_2_-induced apoptosis [12]. MELK also physically interacts with p53 and enhances of p53-dependent apoptosis and cell cycle arrest [32]. Therefore, MELK is considered as a regulator of cell proliferation, differentiation, and apoptosis.

## 3. *MELK* Is Involved in Embryonic Development

Preliminary RT-PCR data have shown that *MELK* is specifically and highly expressed in an ovulated egg and during early mouse development [3]. *MELK* transcripts are barely observed in two-cell stage embryos, but such transcripts are easily detectable in ovulated mouse eggs and increase in later developmental stages. Like other transcripts at the same developmental stage, *MELK* transcripts accumulate from an eight-cell stage to a blastocyst stage [14]. In contrast to *MELK* transcripts, MELK proteins remain detectable throughout pre-implantation development. The presence of MELK protein in the zygote and two-cell stage embryos is likely due to a protein translated from maternal mRNA as observed in E-cadherin [33]. MELK possibly functions as a cytoplasmic serine/threonine kinase to modify other proteins post-translationally at this time, thereby influencing further embryonic development [15].

Further studies have demonstrated that *MELK* is expressed in tissues containing normal progenitor cells [15] and in adult brain progenitor cells [34,35]. Knockdown of a *MELK*-like gene product in zebra fish has indicated that this product may function in primitive hematopoiesis [36].

MELK is associated with anillin, an important cytokinetic protein, and two other proteins co-localized at the equatorial cortex of *Xenopus*. Furthermore, a developmentally regulated transition involving *Xenopus* MELK (xMELK) occurs in cytokinesis during early embryogenesis [16].

## 4. *MELK* in Cancer

Serine/threonine kinases represent a suitable protein class for targeted therapies in cancer. *MELK* overexpression has been detected in various human tumors, suggesting that MELK is a significant contributor to tumorigenesis (Table 1). An increase in *MELK* expression is described in more aggressive forms of astrocytoma, breast cancer, melanoma, and glioblastoma [19,37–39]. Considering the results of preclinical studies, MELK is described as a potential anticancer target in diverse tumor entities [40].

MELK is important in oncogenesis as first emphasized in a previous finding, in which *MELK* expression is increased in tumor-derived progenitor cells and in cancers of non-differentiated cells [40,41,43]. For instance, MELK is required in cells that initiate mammary tumors [44]. *MELK* is also highly expressed in various cell lines established from colorectal carcinoma [45]. Furthermore, MELK is associated with anti-apoptotic activities of breast cancer cells by interacting with Bcl-G, a pro-apoptotic member of the Bcl-2 family [11,46]. Overexpression of MELK suppresses Bcl-G-induced apoptosis, which promotes mammary carcinogenesis [11] and results in poor patient survival in breast cancer and glioblastoma multiforms [19,47]. *MELK* may also function as a therapeutic target associated with the resistance of rectal cancer to chemoradiotherapy [42].

*MELK* knockdown decreases the transformed phenotype of multiple tumor cell lines as determined by *in vivo* xenograft assays as well as *in vitro* proliferation and anchorage-independent growth [40]. Thiazole antibiotic siomycin A is identified as a potent inhibitor of *MELK* expression. The treatment of glioblastoma with siomycin A inhibits tumor growth *in vivo*. In particular, treatment of glioblastoma-derived brain tumor stem cells with siomycin A promotes self-regeneration, decreases invasion, and induces apoptosis [48]. MELK is aberrantly reactivated in cancer stem cells, thereby providing a growth advantage for neoplastic cells and derived tumor progression [44,47]. *MELK* expression in normal progenitor cells indicates that the dysregulation of *MELK* may cause carcinogenesis in various cell types [20].

These data have suggested that the overexpression of *MELK* contributes to the development of tumors, but the inhibition of *MELK* sufficiently affects proliferation and other properties of tumors. Further investigation on *MELK* as a cancer therapeutic target is needed.

## 5. Proteins Interacting with MELK

Preliminary evidence has indicated that MELK is involved in various cellular processes by binding to numerous proteins, thereby resulting in cell cycle regulation, cell proliferation, apoptosis, spliceosome assembly, gene expression, hematopoiesis, embryonic development, and oncogenesis [12,13,36,40,49,50]. Proteins that interact with MELK are listed in Table 2.

MELK can regulate numerous proteins via interaction and phosphorylation. MELK phosphorylates and binds tightly to the zinc finger-like protein 9 (ZPR9), and causes its nuclear accumulation [51,52]. In the nucleus, ZPR9 interacts with the transcription factor v-MYB avian myeloblastosis viral oncogene homolog 2 (B-Myb), a regulator of cell proliferation and differentiation, and enhances its transcriptional activity [52]. MELK also interacts with nuclear inhibitor of protein Ser/Thr phosphatase-1 (NIPP1), a transcription and splicing factor, by binding of a threonine-phosphorylated motif to FHA domain of NIPP1. Furthermore, the MELK-NIPP1 interaction is significantly increased during mitosis, resulting in an inhibition of pre-mRNA splicing [13]. MELK physically interacts with and phosphorylates PDK1 at threonine 354, thereby inhibiting its activity and function. CDC25B is a protein-tyrosine phosphatase that triggers mitosis by activating protein kinase CDK1. MELK can also phosphorylate and interact with CDC25B, by which the G2 accumulation induced by MELK overexpression in cultured cells is counteracted [4]. MELK also physically interacts with p53 *in vivo* and *in vitro*. It phosphorylates Ser15 in the amino-terminal transactivation domain of p53, thereby stimulating p53 activity. This interaction contributes to the enhancement of p53-dependent apoptosis and cell cycle arrest by modulating the stability of p53 [32]. Overexpression of MELK significantly increases the MAPKKK activity of ASK1 by directly interacting with and phosphorylating ASK1, which stimulates the activation of ASK1-mediated signaling to JNK and p38 kinases. Such overexpression of MELK also results in a significant increase in H_2_O_2_-induced apoptosis by interacting with ASK1 [12]. Therefore, the MELK-ASK1 interaction may provide a molecular basis for several proposed MELK functions. Bcl-G, a pro-apoptotic factor, has been the focus of studies recently. It is identified as an important target for MELK, which is associated with resistance to apoptosis [11]. The modification of BCL-G provides an attractive mechanism of the observed pro-survival effects of MELK [11]. MELK positively regulates transforming growth factor-β (TGF-β) transcription by directly interacting with and phosphorylating Smad proteins (Smad2, -3, -4, and -7), which is required for TGF-β-mediated biological functions, such as apoptosis and cell growth arrest [50]. Therefore, these findings provided some support for the regulation of cell cycle, cell proliferation, and apoptosis by MELK. Further, these results may also support the conceptual premise that MELK functions in the development of various tissues by maintaining the balance between cell proliferation and survival.

MELK activity is also regulated via phosphorylation by other proteins. For instance, MELK can be phosphorylated by mitosis-promoting factor (MPF) and MAPK during M phase. Threonine 414, threonine 449, threonine 451, threonine 481, and serine 498 phosphorylation sites in xMELK extract are identified in its egg at the M phase, whereas 14 residues are phosphorylated in recombinant human MELK (hMELK) protein expressed and purified from bacteria. Experiments performed *in vivo* have suggested that xMELK phosphorylation is involved in MPF and MAPK pathways. MPF and MAPK directly phosphorylate xMELK and enhance its kinase activity *in vitro*. In addition, the specific phosphorylation of threonine 449, threonine 451, and threonine 481 in M phase is detected during *Xenopus* oocyte maturation in embryos and in *Xenopus* cultured cells [55].

Thus, many of these interactions result in aberrant signaling involved in cell cycle progression, TGF-β signaling, embryonic development, ASK1-mediated signaling, and apoptosis. Some of these interactions are often ambiguous, and the function of MELK is only partially clear. Further studies should be conducted to identify the exact mechanisms involved and define the function of MELK in normal developmental processes and tumorigenesis.

## 6. Conclusions and Perspectives

Multiple functions of MELK affect numerous proteins and signaling pathways through protein-protein interactions. In general, the results of these interactions are oncogenic. Overexpression of MELK in various human cancers suggests its importance in carcinogenesis. However, the exact function of MELK in oncogenic processes and in other functions remains partially understood. Future studies should determine whether or not MELK contributes to tumorigenesis via a specific interaction or multiple interactions. Furthermore, because protein-protein interactions include direct and indirect interactions, future studies should elucidate whether or not these interactions are dependent on the presence of MELK.

The physiological importance of MELK in cancer is highlighted because of its overexpression in various cancers. However, the molecular mechanism by which MELK is involved in a myriad of protein interactions remains unclear. No particular protein domain has been identified in these proteins that link them with one another. Thus, the activation mechanisms should be elucidated to understand the regulation of the functions of MELK.

Although information about *MELK* remains unclear, its overexpression is involved in cell cycle regulation and cancer development. Further investigation of *MELK* as a therapeutic target may lead to the development of a powerful cancer therapeutic target in various tumors.

## Figures and Tables

**Table 1:** Cancers in which maternal embryonic leucine zipper kinase (MELK) is overexpressed.

Cancer types	Function	References
Pediatric brain tumor	ND	[41]
Colon tumor	Therapeutic target	[40]
Glioblastomas	ND	[37]
Astrocytomas	Therapeutic target to treat human glioblastomas	[39]
Breast cancer	Interacting with Bcl-G and associated with poor prognosis	[11,19]
Melanoma	Mitosis and protein phosphorylation	[38]
Rectal cancer	Contributed to radioresistance and chemoresistance of SNU-503	[42]

Abbreviation: ND, not determined.

**Table 2:** Proteins that interact with MELK.

Protein	Description	Interaction Effect	References
ZPR9	A physiological substrate of MELK kinase *in vivo*	Resulting in the nuclear accumulation of ZPR9	[51,52]
NIPP1	Transcription and splicing factor, inhibitor of protein Ser/Thr phosphatase-1	Regulating cell cycle progression through pre-mRNA processing	[13]
Cdc25B	Protein-tyrosine phosphatase	Inducing cell accumulation in G2	[4]
PDK1	An enzyme responsible for the phosphorylation of the activation loop of Akt/PKB	Inhibiting activity and function of PDK1	[53,54]
P53	Tumor suppressor	Enhancing p53-dependent apoptosis and cell cycle arrest by modulating the stability of p53	[32]
Smad proteins (Smad2, -3, -4, and -7)	Intracellular signaling mediators of the TGF-β signaling pathway	Regulating Smad activities involved in TGF-β signaling	[50]
ASK1	Mitogen-activated protein kinase kinase kinase	Enhancing of JNK-mediated transactivation and H_2_O_2_-induced apoptosis	[12]
Bcl-G	A pro-apoptotic factor	Resistance to apoptosis	[11]
MPF	Mitosis-promoting factor	Phosphorylating MELK and enhancing its kinase activity	[55]
MAPK	Mitogen-activated protein kinase	Phosphorylating MELK and enhancing its kinase activity	[55]
